# Future perspectives of higher standards for trauma teams' organization, support, and evaluation

**DOI:** 10.1007/s00068-022-02196-3

**Published:** 2022-12-21

**Authors:** Oscar E. C. van Maarseveen, Wietske H. W. Ham, Luke P. H. Leenen

**Affiliations:** Department of Trauma Surgery, University Medical Center Utrecht, Heidelberglaan 100, 3584 CX Utrecht, The Netherlands

**Keywords:** Trauma Team, Trauma Resuscitation, Evaluation, Technology, Quality of Care

## Introduction

There is continuous drive to optimize healthcare for severely injured patients. In this article, suggestions are made to introduce higher stands for organization and support and evaluation of trauma teams. Capturing and processing data using artificial intelligence from trauma resuscitations could bring evaluation, reporting, and decision-making to an higher level.

## Evaluation

### Higher standards for evaluation

To obtain a better understanding of the interplay of the complex team and task-based challenges during trauma resuscitation, we propose to use a synchronized data capturing system and analysis platform including machine learning techniques, like the OR black box as described for the OR environment [1]. This system should continuously capture video and audio during trauma resuscitations, as well as patient physiological data, environmental data, and imaging data. In the trauma bay, multiple wall-mounted cameras and microphones should be installed in order to capture team positioning, movements, and communication. Furthermore, sensors and data synchronization of existing vital signs and imaging capturing systems should be implemented. For general performance analysis, all data should be anonymized, synchronized, encrypted, and securely stored. Expert analysts and software-based algorithms should analyze the entire resuscitation process and individual technical and non-technical skills. Only for individual performance evaluation or direct educational purposes, data could be stored without anonymization but temporarily be stored and removed shortly after educational use. This novel evaluation system could play an important role in continued efforts to improve trauma resuscitations, without the need for extended workload of personnel. An advantage of utilizing machine learning techniques is that more data could be analyzed by computers which also reduces the workload of human reviewers.

Although there are currently no reports of institutes using such a system in trauma bay, very similar technology has used to evaluate surgical procedures in the operating room (OR) during surgical procedures and is called the OR black box [1]. Within the last decade, the OR black box has been used to identify distractions, adverse events, and to evaluate teamwork during surgical procedures in the OR. For instance, a recent report of one year’s worth of data using this technology revealed that there were 138 distractions per surgical procedure and a medians of 20 intraoperative mistakes [2]. Another recent study using captured audio, video, and synchronized data of the patient’s vitals found that the non-technical skills of the surgical team could be measured reliably using the non-technical skills for surgeons (NOTSS) behavior marker system [3, 4].

## Support

### Clinical decision support

Strategies to improve trauma teams have traditionally focused on technical and non-technical skills training, as well as retrospective evaluations of their management during specific cases [5–8]. However, these methods do not actively support the trauma team in anticipating adverse events, nor do they help in decision-making during the trauma resuscitation itself. The suggested improved data collecting method, in combination with available artificial intelligence techniques to interpret the data, pave the way for real-time predictions of a patient’s physiological state and decision support. Warnings for upcoming events and real-time treatment suggestions may be given to the trauma team to help them make clinical decisions. An example of how such a system could work was described in the article of Lundberg et al. in 2018 [9], where they revealed an artificial intelligence-based warning system called ‘prescience’ that was able to predict hypoxemia during surgical procedures up to 5 min in advance. This system continuously analyzes vital signs and delivers a risk score to the clinician that updates in real time, along with the rationale for its predictions, which would be extremely useful in a trauma resuscitation.

The human capacity to process multiple variables to predict outcomes is very limited, while computers can process many (almost unlimited) variables at the time. In the study of Halford et al. [9], participants were asked to interpret graphical representations of statistical interactions. In these situations, all independent variables must be processed concurrently. When the subjects processed more variables, their accuracy and speed decreased significantly. According to their findings, the limit of human processing capacity was only four variables the time. We believe that, decision supportive system should not be 'a black box system' that tells us what to do but support us by analyzing the many variables which are forehand during trauma resuscitations.

### Electronic medical records

Electronic medical records aim to improve patient care, but on the other hand, administrative burden in healthcare is a well-known problem, and uncompleted descriptions of trauma resuscitations are common. However, this is due to issues such as the one described in Golob et al. [10] who stated that a busy trauma surgeon has a huge portion of their time dedicated to documentation. During a one-year period, there were 3111 patients admitted to their level one trauma center, and the attending trauma surgeon wrote 26,455 documentation entries which took 1760.5 h to write. Besides the heavy administrative burden, the notes that physicians and nurses write about trauma resuscitations and their findings are retrospectively written, and this form documentation often lacks meaningful details and may even be inaccurate [11].

Thousands of hours of administration could be saved, while improving documentation of trauma resuscitations at the same time. Patients' electronic medical records can be formed by using data input from various sources (such as video, audio, sensors, and existing data capturing of vital signs and imaging) a report of into a patient’s electronic medical records can be made. Recently, Maas et al. [12] describe a system that is potentially able to automate medical reporting, and it consists of three phases that generate an electronic medical report. During the first phase, audiovisual and sensory data, including patient vital parameters, are collected and synchronized. During the second phase, data are interpreted by using and combining speech recognition and motion recognition techniques. In the case of trauma resuscitation, a simple example is that the introduction of an IV line can be detected by using motion recognition. A more advanced example is that the results of auscultation could be detected first by motion recognition (physician listening to the lungs) and speech recognition (physician sharing findings to the rest of the team) while finishing by combining the results. In the third phase, the medical report is generated during a complex process, which, in detail, is described by Maas et al. [12]. In short: Extrapolated text is used to document trauma resuscitations, and the medical report generation is based on a database of the previous reports, ATLS guidelines, and the data gathered during the trauma resuscitation. Artificial intelligence techniques make it possible to combine these elements to a complete an understandable medical documentation.

## Organization

**Fig. 1:**
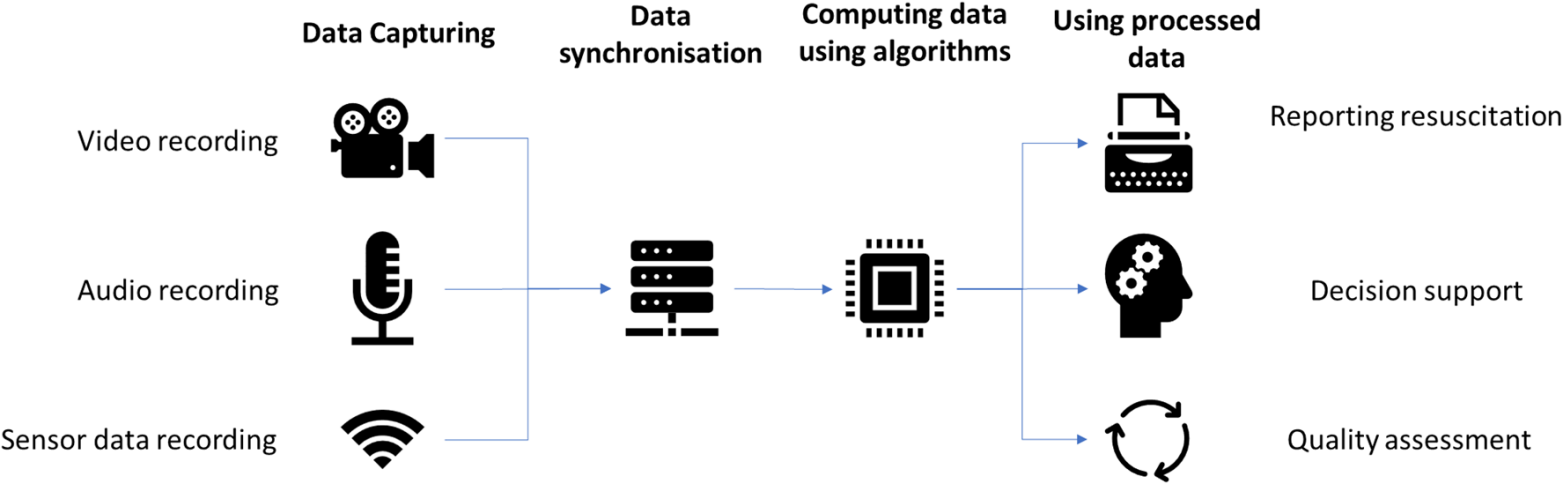
The use of data from technology to improve trauma care

Managing a severely injured patient in the trauma bay poses significant demands on the management process, and multiple specialties are involved in the acute and dynamic setting of trauma resuscitations. The foundation of consistent and good quality handling of these resuscitations is fine-tuned teamwork among these different medical disciplines to achieve a streamlined and simultaneous execution of tasks. Teamwork within trauma teams has gained attention last decades, and it has become widely acknowledged that the non-technical skills of trauma team members are an important part of having and maintaining effective teamwork between physicians, nurses, and ancillary personnel during trauma resuscitation [13–16]. It is critical for every hospital to have a trauma resuscitation algorithm that coordinates among the specialties involved and to practice together on a regular basis in order to engaged trauma team members to be familiar with these algorithms. Nevertheless, most of the focus, when it comes to teamwork, has been on trauma team training, while team composition and staffing have unfortunately received less attention (Fig. 1).

The trauma team leader is an important part of the trauma team and is responsible for the team coordination as well as keeping track of the clinical and logistical implications during the resuscitation [17–19]. With that in mind, experienced trauma team leader may be able to see the big picture and concomitant treatment requirements more quickly than their less experienced colleagues. The previous studies have shown that a more experienced trauma team leader speeds up the pace of trauma resuscitations and could lead to a reduction on mortality [20, 21]. Experienced trauma team leaders are most beneficial for patients requiring fast intervention to treat acute life threatening injuries such as a severe hemorrhage [22]. Therefore, trauma centers should consider having a trauma team that includes an experienced trauma team leader in cases where a resuscitation with a high pace is desired, which is typically the resuscitation of the most severely injured patients. For every trauma center, resuscitation of the most severely injured patients by experienced trauma team leaders is likely beneficial for patient outcome; however, it is intuitively more feasible to organize a staff occupation with enough experienced surgeons at larger volume trauma centers. Besides the benefits, an experienced trauma team leader brings during the trauma resuscitation, their additional experience for managing the severely injured may also have beneficial implications for the process after the resuscitation in the trauma bay, such as time to the operation room or additional imaging. For the management pace of severely injured patients, a recent systematic review showed that ten out of sixteen included studies found at least one process-related outcome was improved after implementing an in-house attendance policy for trauma surgeons instead of the on-call attendance policy during which less experienced surgical residents act as the house officer [23].

Finally, staffing variation should get more attention as the nature and consequences of staffing changes within trauma teams are understudied. The previous studies showed that familiarity between team members improved results for teamwork, processes, or patient care in trauma teams during simulated resuscitations [24], vascular surgical teams [25], abdominal surgical teams [26], and gynecological surgical teams [27]. Trauma teams may benefit even more from familiarity, since, in contrast to predictable circumstances during elective surgery or simulated environments, the circumstances during severe injury resuscitations are more stressful and unpredictable, necessitating highly adaptive teams that could rely on their team members. More familiarity within teams could be achieved by less staffing variation within teams. This could be achieved by forming literally forming multiple trauma teams within one trauma center. We propose that each team's core group should be composed of a limited number of team members and should vary relatively little over time. The core group of team members should consist of members who accomplish the majority of the actions and/or bear most of the responsibility during trauma resuscitations. Finally, the team members of the same team should then be scheduled concurrently.
